# Fecal carriage of extended-spectrum beta-lactamase (ESBL)-producing *Escherichia coli* in South American camelids and biosecurity practices among farms in northern Italy

**DOI:** 10.1007/s11259-025-10653-8

**Published:** 2025-01-23

**Authors:** Laura Filippone Pavesi, Maria Cristina Rapi, Martina Penati, Laura Musa, Federica Santandrea, Vincenzo Ferrulli, Ilaria Martucci, Antonio Boccardo, Guido Grilli, Maria Filippa Addis, Valerio Bronzo

**Affiliations:** 1 Department of Veterinary Medicine and Animal Sciences, University of Milan, Lodi, 26900 Italy; 2 Laboratorio di Malattie Infettive degli Animali - MiLab, University of Milan, Lodi, Italy

**Keywords:** South American camelids, ESBL, Biosecurity, Antimicrobial resistance

## Abstract

South American camelids (SACs), particularly llamas (*Lama glama*) and alpacas (*Vicugna pacos*) are gaining popularity in Europe. Initially valued for their fiber and land management capabilities, these animals are now also kept for animal therapy, outdoor activities, and as companion animals. Despite their close interactions with humans and other animals, there is limited research on the transmission of microbes or antimicrobial resistance genes from SACs. This study aimed to survey the fecal carriage of extended-spectrum beta-lactamase ESBL-producing *Escherichia coli* (ESBL*- E. coli)* in SACs. A questionnaire was administered on-site to the farmers to survey management and biosecurity measures. Twelve farms from northern Italy (Lombardy, Piedmont, Veneto, and Emilia-Romagna) participated in the study. Fecal samples were analyzed to identify ESBL- *E. coli* and subjected to bacteriological culture on CHROMagar™ ESBL plates. Isolate identification was accomplished by MALDI-TOF MS, then subjected to the double-disk synergy test (DDST) and examined for 3 ESBL-encoding genes (*bla*_CTX−M_, *bla*_TEM_, *bla*_SHV_) via PCR analysis. A total of 125 SACs (19 llamas and 106 alpacas) were included. Four (3.2%) of these animals were positive for ESBL- *E. coli*. Two isolates carried the *bla*_CTX−M_ gene; one had both *bla*_CTX−M_ and *bla*_TEM_, and one was negative for all ESBL-encoding genes. Furthermore, none of the ESBL- *E. coli* isolates tested positive for the *bla*_SHV_ gene. Several data from our questionnaire revealed a lack of biosecurity protocols, which aligns with other studies. The prevalence of ESBL-producing bacteria identified in this study was lower than in different other studies. Despite the low biosecurity levels observed on the SAC farms, our findings showed a low occurrence of ESBL- *E. coli* and a low carriage rate of multidrug-resistant (MDR) ESBL- *E. coli*.

**Supplementary Information:**

The online version contains supplementary material available at 10.1007/s11259-025-10653-8.

## Introduction

South American camelids (SACs), particularly llamas (*Lama glama*) and alpacas *(Vicugna pacos)*, are becoming increasingly popular in Europe (Neubert et al. 2021; Gonzalez-Santamarina et al. 2022a; Sala et al. 2024). Among the various uses of SACs, the most prominent ones include producing hypoallergenic fiber, landscape management, and using their manure for natural soil fertilization (Frank et al. 2006). More recently, SACs gained new roles in animal-assisted therapy and outdoor activities such as trekking. They are also increasingly kept as companion animals (Lambacher et al. 2015; Gonzalez-Santamarina et al. 2022a). Due to their close interactions with humans and other animals, SACs can serve as carriers for infectious agents, potentially leading to the shedding of zoonotic, such as *Salmonella* sp. and *Escherichia coli*, and non-zoonotic pathogens (Rahimi et al. 2012; Fadlelmula et al. 2016; Konieczny and Pomorska-Mól 2024). The diversity of pathogens harbored by SACs poses significant challenges for disease surveillance and control, attributed partly to the relative resistance of camelids to many ruminant diseases, so that they may serve as pathogen reservoirs for other ruminant species (Halsby et al. 2017). Furthermore, biosecurity measures are weaker compared to other livestock farming (Barrington et al. 2006; Jones and Boileau 2009). Studies have highlighted the presence of antimicrobial-resistant bacteria in SAC populations, including strains resistant to commonly used antibiotics like tetracyclines and beta-lactams (Luna E. et al. 2012; Barrios-Arpi et al. 2016; De la Cruz Carhuapoma et al. 2020). In recent years, the attention on the epidemiology of multidrug-resistant (MDR) bacteria in both human and veterinary medicine has increased, with the focus on the emergence of extended-spectrum β-lactamase (ESBL)-producing *Enterobacteriaceae* (Poirel et al. 2018). Special attention is given to ESBL-producing *Escherichia coli* (ESBL- *E. coli*) within the *Enterobacteriaceae* family. *E. coli* is known to be one of the bacterial species in which the selection of resistance genes has occurred more rapidly following the widespread use of antimicrobials. This condition led to the recognition of E. coli as an AMR surveillance indicator and a significant reservoir of resistance genes co-circulated by this bacterium between the environment, animals, and humans (Tadesse et al. 2012; Chung et al. 2023). Acquiring the ability to synthesize ESBL enzymes by *E. coli* results in additional resistance to third- and fourth-generation cephalosporins, defining a severe threat to human and animal health (Gonggrijp et al. 2016). This study aimed to evaluate the presence of ESBL- *E. coli* in SACs in Northern Italy, considering their potential role as carriers of antimicrobial resistance from a One Health perspective. In addition, a secondary purpose of this study was to describe alpaca management practices on farms registered by the National Alpaca and Llama Society (SNAEL) in northern Italy with particular emphasis on herd biosecurity information through an interview-based questionnaire.

## Materials and methods

### Study design and sample size

Following STROBE guidelines (von Elm et al. 2008), we conducted an observational study using data from South American Camelids farms in Northern Italy. Based on the 31% expected prevalence of ESBL- *E. coli* (Gonzalez-Santamarina et al. 2022b), using a 95% confidence level and an absolute precision of 10%, 82 SACs must be enrolled. The sample size was calculated using the formula published by Daniel (1999) for epidemiological prevalence studies.

Additionally, the questionnaire assessing the structural and managerial characteristics of the farms was developed based on guidelines from the Consensus-Based Checklist for Reporting of Survey Studies (CROSS) (Sharma et al. 2021) (Supplementary File 1).

## Herds enrollment and questionnaire

The farms included in this study were chosen based on the availability of breeders registered with the National Alpaca and Llama Society (SNAEL) after contacting them by email to describe the purpose of the study and the presence of at least one SAC in the farm. Thirty-four farms were registered at SNAEL. For convenience, we chose farms no more than 300 km from the University of Milan, which responded to our email between February 10 and February 20, 2024. Sampling was conducted from late February to March 15, 2024. A questionnaire was completed on-site, and an interview with the owners was conducted. The first author administered the questionnaire in the Italian version (Supplementary File 2) after signing a declaration of consent for taking samples and analyzing them for scientific reasons and to ensure confidentiality. To ensure confidentiality and anonymity, all the names of the farms were substituted with numbers. The survey was designed to minimize non-response bias and missing data by using Italian, incorporating a limited number of questions, and employing 45 multiple-choice questions. The questionnaire was based on a previous one developed by Gonzalez-Santamarina and colleagues (Gonzalez-Santamarina et al. 2022a) and was used to assess the management and biosecurity practices of SACs breeding. Because it was based on a previous questionnaire, we did not do any pre-tests of the questionnaire. The form was divided into sections: (I) Farm organization (II) Interactions with other animals and people. (III) Transport of animals and quarantine; (IV) Adult SACs and reproduction management; (V) Health management; (VI) Calving management and cria rearing; (VII) Antimicrobial treatments.

The results were compiled in an Excel spreadsheet database. The response rate was determined based on the methodology outlined by Dillman et al. (2014). Missing data from the questionnaire were analyzed following Rubin’s approach (1987). Additionally, a descriptive analysis of the questionnaire responses was conducted.

## Animals, sample collection, and ethics statement

All the enrolled farms were visited only once. All SACs on each farm were enrolled and identified, and a clinical examination was conducted on each animal. The species, breed, sex, age, and current health status or prior antimicrobial treatment were recorded. For each enrolled animal, fecal samples were collected from the rectal ampoule, transported to the laboratory, stored at 5 °C, and processed within 24 h after collection. The study was conducted under the Declaration of Helsinki and approved by the Institutional Committee for Animal Welfare of the University of Milan (Protocol number 51_2024).

## Isolation and characterization of ESBL- *E. Coli*

Extended-Spectrum Beta-Lactamase-Producing - E. coli was identified as described by Penati et al. (2024). At least three colonies indicating ESBL- E. coli on CHROMagarTM ESBL plates were selected for species identification using the MBT Microflex LT/SH MALDI-TOF mass spectrometer (Bruker Daltonik GmbH, Bremen, Germany) following the protocol by Rosa et al. (2022). Colonies retrieved from CHROMagarTM ESBL plates were sub-cultured on blood agar plates (Microbiol, Cagliari, Italy) and subjected to ESBL phenotyping using the double-disk synergy test (DDST). Following EUCAST guidelines, DDST was performed using ceftazidime (30 µg), cefotaxime (30 µg) discs, and amoxicillin/clavulanic acid (30 µg) discs (EUCAST, 2017).

## Antimicrobial susceptibility testing

The isolated strains underwent antimicrobial susceptibility testing (AST) using the disk diffusion method outlined in the literature (Luna et al. 2012; Barrios-Arpi et al. 2016; CLSI 2024a). A panel of nine antimicrobials from six different classes was employed. This panel, also used in other studies on these species (Luna et al. 2012; Barrios-Arpi et al. 2016), included amoxicillin/clavulanic acid (30 µg), ampicillin (10 µg), ceftiofur (30 µg), enrofloxacin (5 µg), tetracycline (30 µg), gentamicin (10 µg), cefazolin (30 µg), florfenicol (30 µg), and trimethoprim/sulfamethoxazole (25 µg). Antibiotic susceptibility was determined by measuring the zone of inhibition following CLSI and EUCAST breakpoints. Without specific CLSI breakpoints for SACs, those defined for cattle and humans were used (CLSI 2024a, CLSI 2024b; EUCAST 2024). Isolated strains resistant to three or more antimicrobial classes were considered MDR strains, according to Magiorakos et al. (2012).

### Characterization of ESBL-encoding genes

Genomic DNA was extracted from ESBL- E. coli isolates using the DNeasy Blood & Tissue Kit (QIAGEN Diagnostics GmbH, Germany) with some modifications starting from step 2 (Cremonesi et al. 2006). The amount and quality of DNA were measured by NanoDrop ND-1000 spectrophotometer (NanoDrop Technologies, Wilmington, DE). All ESBL- E. coli isolates were screened for blaCTX-M and blaTEM genes by Multiplex PCR and blaSHV gene by standard PCR, as described by (Monstein et al. 2007). All amplified PCR fragments were visualized by 2% agarose gel electrophoresis (GellyPhor, Euroclone, Milan, Italy), stained with MIDORI Green Advance (4–6 ul/100 ml; Nippon Genetics Europe GmbH, Germany), and visualized by iBrightTM CL1500 Imaging System (Thermo Fischer Scientific, Life Technologies Corporation, Singapore). A 100-bp DNA ladder (Finnzymes, Espoo, Finland) was included in each gel.

## Results

### Enrolled herds

Twelve farms from different northern Italian regions, including Lombardy (6 farms), Piedmont (3 farms), Veneto (2 farms), and Emilia-Romagna (1 farm), were identified and enrolled. Table 1 describes the number of samples per farm, animal species bred, and geographic location of each enrolled farm. All the farms that were contacted participated in the study.

**Table 1:** Number of samples collected (*N* = 125), geographic location and species of SACs reared on each enrolled farm

Farm No.	No. of samples	Region	Province	Animal species
1	3	Lombardy	Bergamo	Alpaca
2	9	Lombardy	Bergamo	Llama
3	15	Lombardy	Bergamo	Alpaca
4	1	Lombardy	Cremona	Alpaca
5	20	Piedmont	Torino	Alpaca
6	17	Piedmont	Torino	Alpaca and Llama
7	21	Veneto	Treviso	Alpaca
8	11	Veneto	Treviso	Alpaca
9	5	Lombardy	Lodi	Llama and Alpaca
10	16	Emilia-Romagna	Piacenza	Alpaca
11	1	Piedmont	Novara	Alpaca
12	6	Lombardy	Milan	Alpaca

### Questionnaire

Based on the questionnaire responses (Supplementary File 3), all farmers (12 out of 12) completed the questions, resulting in a 100% response rate with no missing data. Of the twelve farms considered, nine bred only alpacas (Vicugna pacos), one reared only llamas (Lama glama), and two bred both species. SACs were used for fiber production (5 out of 12 farms), landscape management (4 out of 12 farms), trekking (4 out of 12), kept as a hobby (2 out of 12), and used for animal-assisted therapy (1 out of 12). Almost all farms (10 out of 12) purchase animals frequently and keep the SACs in groups. Eleven out of twelve farms kept other animal species on the farm, and three farms prevented them from directly contacting their SACs.

The people who interact with SACs are the family members (12 out of 12 farms), visitors (5 out of 12), farm personnel (2 out of 12), and veterinarians (2 out of 12). Three out of twelve farms do not breed their animals, while seven have established breeding programs within their herds. In two farms, breeding programs may involve bringing the stallion to the herd or breeding with other farms. In these nine farms, the calving always happened in the herds, and six out of nine farms had a monitoring of calving. Of these six farms, only five were checked for malformation, and the external umbilical structures were disinfected at birth. In the other four farms, none of these procedures were carried out. The veterinarian needed to wear dedicated clothing and footwear in only two out of twelve farms, but not other external visitors. As for antimicrobial use, it correlates with farm health problems. Half the twelve farms surveyed reported no infectious disease cases and did not employ antimicrobials. Six farms have used antimicrobials; three used antimicrobials in the previous year. The primary health issues related to antimicrobial use in these herds over the past year included *Candidatus Mycoplasma haemolamae* (in 2 out of 3 farms), treated with oxytetracycline, and diarrhea on one farm, addressed with third-generation cephalosporins. Additionally, penicillin was administered in the remaining three herds for dental or dermatological problems, but not within the past year.

### Bacteriological culture results

A total of 125 SACs (19 llamas and 106 alpacas), ranging in age from 2 months to 12 years old, were included. Of these, 4 (3.2%) animals tested positive for ESBL- *E. coli*. Specifically, these were isolated from the feces of one adult alpaca and one cria from a farm in Piedmont, one adult alpaca from a farm in Lombardy, and one cria from a farm in Emilia-Romagna. The remaining isolates identified by MALDI-TOF MS belonged to other genera; the most frequently identified were *Pseudomonas* spp., *Acinetobacter* spp., and *Achromobacter* spp. (Supplementary File 4).

### Antimicrobial susceptibility testing of ESBL- *E. Coli*

**Fig. 1:**
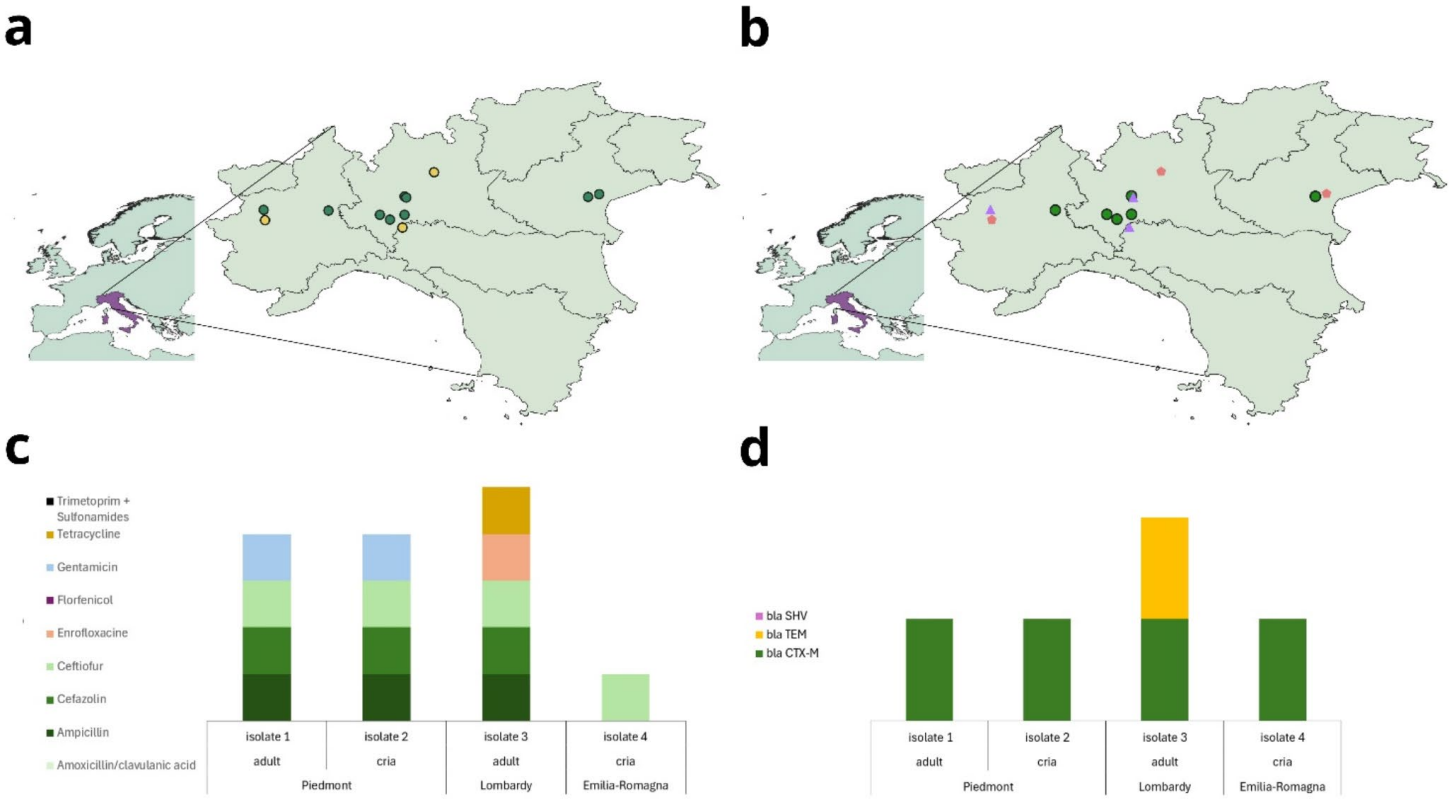
(**a**) Distribution of ESBL-*E. coli* positive (yellow dots) and negative (green dots) farms; (**b**) distribution of herds where antimicrobial treatments were administered within one year before sampling (purple triangles), herds where antimicrobials were administered more than one year before sampling (pink pentagons) and herds where no antimicrobials were used (green dots); (**c**) antimicrobial resistance profiles of the ESBL- *E. coli* isolates determined by disc diffusion test; (**d**) ESBL encoding genes tested by PCR for the ESBL- *E. coli*. The maps were created using QGIS

All *E. coli* isolates identified as suspected ESBL producers at the bacteriological culture phase were also positive for the DDST, confirming the production of ESBL. Susceptibility to the carbapenem class was observed in all ESBL isolates. Based on the antimicrobial susceptibility test results of ESBL- *E. coli* (Fig. 1), the highest resistance level was observed for ceftiofur, a β-lactam, with all isolates resistant to it (100%). Concerning other β-lactams, 75% of isolates were resistant to ampicillin and cefazolin. Regarding fluoroquinolones, 75% of isolates were susceptible to enrofloxacin and for amphenicols, 100% of isolates were susceptible. Concerning aminoglycosides, 50% of isolates were resistant to gentamicin. For the tetracycline class, 75% of isolates were susceptible. Concerning sulfonamides, 100% of the isolates were susceptible to trimethoprim/sulfamethoxazole. As a result of the resistance patterns observed, three ESBL- *E. coli* resistance profiles were identified, with one classified as MDR. Profile 1 was found in two animals (one adult and one cria from Piedmont), profile 2 was found in one animal and showed MDR (one adult from Lombardy), and profile 3 was found in one animal (one cria from Emilia-Romagna).

### Characterization of ESBL-encoding genes

Two isolates from Piedmont tested positive only for the *bla*_CTX−M_ gene, while the ESBL- *E. coli* isolate from Lombardy was positive for both *bla*_CTX−M_ and *bla*_TEM_ genes. The ESBL- *E. coli* isolate from Emilia-Romagna tested negative for the ESBL-encoding genes. All ESBL- *E. coli* isolates were negative for the *bla*_SHV_ gene.

## Discussion

In the last decade, the SAC population and farms have notably increased in Europe and Italy (Neubert et al. 2021; Sala et al. 2024). Given the new trend of breeding SACs for activities with people and considering the frequent interactions that these animals have, it is crucial to collect data related to the infectious diseases affecting these animals, their breeding conditions, biosecurity, and the prevalence of antibiotic resistance in the bacteria harbored in their gut (Gonzalez Santamarina et al. 2022a). To date, limited data regarding the presence of ESBL- E. coli strains and their AMR patterns from SACs are available. To our knowledge, this is the first study that assesses the occurrence of ESBL- E. coli from single fecal samples from SAC in Northern Italy. According to the questionnaire, most farms applied the same biosecurity measures. Eleven out of twelve farms raise SACs for activities with humans, and five have visitors outside the owners’ families, aligning with the emerging trend of SAC farming in Europe (Neubert et al. 2021; Gonzalez-Santamarina et al. 2022a). The farms with more than one SAC usually kept them in groups, as shown in Supplementary File 3. Several data from our questionnaire revealed a lack of some external biosecurity protocols, such as the absence of quarantine measures before introducing new animals and the frequent avoidance of dedicated clothing and footwear for visitors (Neubert et al. 2021; Gonzalez-Santamarina et al. 2022a). Another finding regarding the biosecurity is the possibility of interaction between SACs and other animal species, such as dogs, cats, poultry, or occasionally wildlife (8 out of 12 farms) due to the lack of physical barriers, as opposed to the situation reported by Neubert and colleagues (Neubert et al. 2021). These gaps in biosecurity measures in the enrolled herds are a concern, as these inadequacies could facilitate the spread of AMR bacteria and genes (Graham et al. 2019). Antimicrobial use is generally low or nonexistent in these farms, except for significant health issues like *Candidatus* M. haemolamae, which are treated with oxytetracycline (Barrington et al. 1997).

The study found that only four out of 125 (3.2%) fecal samples were positive for ESBL- *E. coli*, likely due to these farms’ low use of antimicrobials, particularly β-lactams (Hund et al. 2023). Two of the three farms with confirmed ESBL- *E. coli* were dealing with or had recently dealt with *Candidatus* M. haemolamae and had used oxytetracycline for treatment. Regarding the low consumption of antimicrobials, we identified only one MDR E. coli strain with resistance to ampicillin, cefazolin, ceftiofur, enrofloxacin, and tetracycline while testing nine antimicrobial molecules that are the most used for the treatment in these species. The resistance patterns of the other ESBL- *E. coli* are comparable to others present in the literature (Luna et al. 2012; Barrios-Arpi et al. 2016), and the prevalence of ESBL- *E. coli* is lower compared to other European studies (Gonzalez-Santamarina et al. 2022a). The prevalence of ESBL—*E. coli* in adult alpacas and crias in this study is different from that in other livestock species, especially cattle, where ESBL—*E. coli* shows a higher prevalence in calves’ feces compared to adult animals (Homeier-Bachmann et al. 2022; Penati et al. 2024), but in this study, the ESBL- *E. coli* was found both in adult and cria alpacas.

The MDR strain was found on a farm where penicillin had been used to treat severe sarcoptic-mange-related pyodermas. Given that some antimicrobials are considered critical to Public Health and that the global spread of MDR *E. coli* is of significant concern, it becomes essential to consider the low prevalence in this study of MDR-ESBL-producing *E. coli* (Benavides et al. 2024). Three of the four isolates tested were confirmed as ESBL- *E. coli*, with blaCTX-M identified as the predominant ESBL-encoding gene, as described previously (Gonzalez-Santamarina et al. 2022a; Gonzalez-Santamarina et al. 2022b). One *E. coli* isolate, despite being phenotypically positive for ESBL, resulted negative for the genes tested.

While this is an unusual observation, this phenotype could be result of other resistance mechanisms, such as mutations in porin proteins or efflux pumps (Christaki et al. 2020). Outer membrane permeability could be specifically altered against larger molecules like third-generation cephalosporins while still allowing smaller molecules like aminopenicillins to enter, some efflux pumps may be more effective against third-generation cephalosporins compared to aminopenicillins or first-generation cephalosporins (Nikaido and Pagès 2012).

Regarding the weaknesses of this research, our study has some limitations, including using a convenience sample with varying herd and animal numbers per region and testing only the antimicrobial resistance of ESBL- *E. coli* without using genomic sequencing techniques. Another study limitation was the absence of statistical analysis of the questionnaire responses and their correlation with the bacteriology results.

Despite this, the findings emphasize the presence of antimicrobial resistance regardless of limited antimicrobial use, underscoring the need for surveillance and biosecurity measures in low antimicrobial consumption farming.

## Electronic supplementary material

Below is the link to the electronic supplementary material.

Supplementary Material 1

Supplementary Material 2

Supplementary Material 3

Supplementary Material 4

## Data Availability

No datasets were generated or analysed during the current study.
