# Supplementary material for: Fecal carriage of extended-spectrum beta-lactamase (ESBL)-producing Escherichia coli in South American camelids and biosecurity practices among farms in northern Italy
Source: Vet Res Commun. 2025 Jan 23;49(2):88. doi: 10.1007/s11259-025-10653-8 (PMC11757900; doi:10.1007/s11259-025-10653-8)
Supplement: Supplementary file 1 — Supplementary Material 1 [file 11259_2025_10653_MOESM1_ESM.docx]

**Questionnaire Biosecurity and Management of South American Camelids**

**General information:**

Name:

Address:

Email address:

Date:

1. **General questions about farm organization:**

Do you run this farm as:

- Main occupation
- Sideline
- Hobby farming

For what purpose are South American camelids bred:

- Wool production
- Animal-assisted intervention
- Breeding
- Trekking and events
- Landscape management
- Other (specify___________________________________________)

Which species do you breed?

- Alpacas
- Llamas
- Both

How many South American camelids are present in the herd?

- Mare,
- Crias,
- Stallions
- Total

Are these groups stable in their composition?

- A single group
- Female animals with crias
- Male animals in one group
- A group with only young animals
- Other (specify___________________________________________)

1. **Interactions with other animals and people:**

Are there any other animal species on the farm?

- Yes
- No

If yes, specify the species: ________________________________________

Do these animals have direct contact with South American camelids?

- Yes
- No

Are there socializations with other species?

- Yes
- No

Are there other farms within a 3 km radius?

- Yes
- No

Which people have on-site contact with South American camelids?

- - Family members
  - Farm personnel
  - Veterinarian
  - Visitors

Do the animals have contact with people in other places?

- Yes
- No

If yes, specify where: __________________________________________

Do the regular people have contact with additional animals?

- Yes, to ________________________________________________
- No

1. **Transport of animals and quarantine procedures**

Do you buy new animals regularly?

- Yes, frequency __________________________________________
- No

Where do purchased animals come from?

- Italy
- Foreign countries (EU)
- Foreign third countries

Are animals sold regularly?

- Yes, frequency
- No

Is the keeping of the stallion outsourced?

- Yes
- No

Do you perform quarantine for purchased animals?

- Yes
- No

Do you perform quarantine for animals coming back from the mating season?

- Yes
- No

Do you have a quarantine pen?

- Yes
- No

1. **Adult SACs and reproduction management**

Are the animals used for reproduction /breeding?

- Yes
- No

Where are the animals mated?

- In the own herd
- In the farm's own stallion farm
- In an external animal husbandry
- The external stallion is moved to the own herd

Are there/have there been abortions on the farm?

- Yes, frequency
- No

1. **Health management in the infirmary and management of outbreaks**

Are there one or more problems in the herd from the following list?

- *Candidatus* Mycoplasma haemolamae
- Respiratory diseases
- Neurological diseases
- Reproduction problems
- Dental problems
- Arthritis (especially in young animals)
- Diarrhoea
- Dermatologic problems

Do you vaccinate?

- Yes, for: __________________________________________________
- No

Is it mandatory to wear dedicated clothing and footwear to access to the farm?

- Always
- Sometimes _____________________________________________
- Never

Is there an infirmary pen?

- Yes
- No

1. **Calving pen management and cria rearing:**

Where does the birth take place?

- In the herd
- The pregnant animal is separated for the birth

Is there a monitoring for the calving?

- Yes
- No

Is special cleaning done after birth?

- Mucked out
- Cleaned
- Disinfected
- No

Do you check for malformation (atresia ani or cleft palate)?

- Yes
- No

Do you check if the cria drinks enough colostrum?

- Yes
- No

Are there/were there weak young animals on the farm?

- Yes
- No

Have any young animals died within the first 14 days after birth?

- - Yes, specify causes ______________________________________
  - No

1. **Antimicrobial treatment**

How is the consumption of antibiotics on the farm estimated?

- Low
- Medium
- High
- Extremely high

Have antibiotics been used in the last year? How often?

- Yes, ___________________________________________________
- No

Which antibiotics did you use in the last 12 months?

___________________________________________________

Did you do a microbiology investigation with or without an antibiotic susceptibility test before treating animals with antimicrobials?

- Yes, without antibiotic susceptibility test
- Yes, with antibiotic susceptibility test
- No
- Antibiotics were started only after the results of the antibiotic susceptibility test

How were the antibiotics administered?

- Orally
- Injection under the skin
- Injection into the muscles
- Injection in the vein

How many animals do you usually treat?

- Single animal treatment
- Group treatment

For how long were the antibiotics administered?

- 1-2 days
- 3-5 days
- 6-7 days
- 8-14 days
- Longer

How are antibiotic-treated animals kept?

- In the normal group
- Individually
- Separated with other antibiotic-treated animals.

Are there available antibiotics on the farm?

- Yes
- No

Are antibiotics used without consulting a veterinarian?

- Yes
- No

**Questionario Biosicurezza e gestione dei camelidi sudamericani**

**Informazioni generali:**

**Nome:**

**Indirizzo:**

**Indirizzo e-mail:**

**Data:**

**1.** **Domande generali sull'organizzazione dell'azienda agricola:**

Gestite l'azienda come:

o Occupazione principale

o Occupazione secondaria

o Agricoltura per hobby

A quale scopo vengono allevati i camelidi sudamericani:

o Produzione di lana

o Intervento assistito con animali

o Allevamento

o Trekking ed eventi

o Gestione del territorio

o Altro (specificare__________________________________________)

Quali specie allevate?

o Alpaca

o Llama

o Entrambi

Quanti camelidi sudamericani sono presenti nella mandria?

o Femmine adulte,

o Cria,

o Stalloni

o Totale

Questi gruppi sono stabili nella loro composizione?

o Un singolo gruppo

o Femmine con cria

o Animali maschi in un gruppo

o Un gruppo con soli animali giovani

o Altro (specificare___________________________________________)

**2.** **Interazioni con altri animali e persone:**

Ci sono altre specie animali nell’azienda?

o Sì

o No

Se sì, specificare la specie: ________________________________________

Questi animali hanno contatti diretti con i camelidi sudamericani?

o Sì

o No

Esiste una socializzazione con altre specie?

o Sì

o No

Ci sono altre aziende agricole nel raggio di 3 km?

o Sì

o No

Quali persone hanno contatti con i camelidi sudamericani?

o Membri della famiglia

o Personale dell'azienda agricola

o Veterinario

o Visitatori

Gli animali hanno contatti con persone in altri luoghi?

o Sì

o No

Se sì, specificare dove: __________________________________________

Le persone abituali hanno contatti con altri animali?

o Sì, a ________________________________________________

o No

**3.** **Trasporto di animali e procedure di quarantena**

Acquistate regolarmente nuovi animali?

o Sì, frequenza __________________________________________

o No

Da dove provengono gli animali acquistati?

o Italia

o Paesi esteri (UE)

o Paesi terzi

Gli animali vengono venduti regolarmente?

o Sì, frequenza

o No

Gli stalloni stanno in un luogo diverso dagli altri?

o Sì

o No

Eseguite la quarantena per gli animali acquistati?

o Sì

o No

Eseguite la quarantena per gli animali che tornano dalla stagione della monta?

o Sì

o No

Avete un recinto di quarantena?

o Sì

o No

**4. SAC adulti e gestione della riproduzione**

Gli animali sono utilizzati per la riproduzione?

o Sì

o No

Dove avviene il programma di accopiamento gli animali?

o Nel proprio allevamento

o Nell'allevamento degli stalloni

o In un allevamento esterno

o Lo stallone esterno viene trasferito nella propria mandria

Ci sono o ci sono stati aborti nell'azienda?

o Sì, frequenza

o No

**5. Gestione della salute in infermeria e gestione delle epidemie**

Ci sono uno o più problemi nella mandria tra quelli elencati di seguito?

o *Candidatus* *Mycoplasma haemolamae*

o Malattie respiratorie

o Malattie neurologiche

o Problemi di riproduzione

o Problemi dentali

o Artrite (soprattutto negli animali giovani)

o Diarrea

o Problemi dermatologici

Vaccinate?

o Sì, per: __________________________________________________

o No

È obbligatorio indossare abbigliamento e calzature dedicate per accedere all'azienda?

o Sempre

o A volte _____________________________________________

o Mai

C'è una penna per l'infermeria?

o Sì

o No

**6. Gestione del parto e dei cria:**

Dove avviene il parto?

o Nel gruppo

o L'animale gravido viene separato per il parto

Esiste un monitoraggio per il parto?

o Sì

o No

Viene effettuata una pulizia speciale dopo la nascita?

o Ripulito

o Pulito

o Disinfettato

o No

Controllate se ci sono malformazioni (atresia ani o palatoschisi)?

o Sì

o No

Controllate se il cria assuma abbastanza colostro?

o Sì

o No

Ci sono/ci sono stati animali giovani e deboli nell’azienda?

o Sì

o No

Cria sono morti entro i primi 14 giorni dalla nascita?

o Sì, specificare le cause ______________________________________

o No

**7. Trattamento antimicrobico**

Come viene stimato il consumo di antimicrobici nell'azienda agricola?

o Basso

o Medio

o Alto

o Estremamente alto

Sono stati utilizzati antimicrobici nell'ultimo anno? Con quale frequenza?

o Sì, ___________________________________________________

o No

Quali antibiotici ha utilizzato negli ultimi 12 mesi?

___________________________________________________

Avete effettuato un'indagine microbiologica con o senza test di suscettibilità agli antibiotici prima di trattare gli animali con antimicrobici?

o Sì, senza test di suscettibilità agli antimicrobici

o Sì, con test di suscettibilità agli antimicrobici

o No

o Gli antibiotici sono stati somministrati solo dopo i risultati del test di suscettibilità antibiotica.

Come sono stati somministrati gli antimicrobici?

o Oralmente

o Iniezione sottocutanea

o Iniezione intramuscolare

o Iniezione endovenosa

Quanti animali trattate di solito?

o Trattamento di un singolo animale

o Trattamento di gruppo

Per quanto tempo sono stati somministrati gli antibiotici?

o 1-2 giorni

o 3-5 giorni

o 6-7 giorni

o 8-14 giorni

o Più lungo

Come vengono tenuti gli animali trattati con antimicrobici?

o Nel gruppo normale

o Individualmente

o Separati con altri animali trattati con antibiotici.

Sono disponibili antibiotici nell'azienda?

o Sì

o No

Vengono utilizzati antimicrobici senza consultare un veterinario?

o Sì

o No
